# A Novel Role for Niemann-Pick Disease Type 2C Protein in Papillae Formation

**DOI:** 10.1371/journal.pone.0015777

**Published:** 2011-01-06

**Authors:** Masahiro Sugawara, Hidemi Ohye, Chisato Tomoda, Takahiko Kogai, Yoshimasa Kamata, Gholam H. Pezeshkpour, Farhad Moatamed

**Affiliations:** 1 Endocrinology and Diabetes Division, Department of Medicine, Veterans Affairs Greater Los Angeles Health Care System, David Geffen School of Medicine at University of California Los Angeles, Los Angeles, California, United States of America; 2 Primary Care Service, Department of Medicine, Veterans Affairs Greater Los Angeles Health Care System, David Geffen School of Medicine, University of California Los Angeles, Los Angeles, California, United States of America; 3 Department of Pathology, Hirosaki University School of Medicine, Hirosaki, Japan; 4 Department of Pathology, Veterans Affairs Greater Los Angeles Health Care System, David Geffen School of Medicine, University of California Los Angeles, Los Angeles, California, United States of America; University of South Florida College of Medicine, United States of America

## Abstract

**Background:**

Despite the presence of papillary structures and papillary tumors in humans, the mechanism of papillae formation is unknown. We describe herein a novel role for Niemann-Pick disease type 2C (NPC2) protein, a cholesterol binding protein in the lysosome, in papillae formation.

**Methodology/Principal Finding:**

We examined NPC2 protein expression in surgical samples of papillary tissues by immunohistochemical stain, and all papillary tissues expressed NPC2 protein in the epithelium. To examine our hypothesis of NPC2 protein-mediated papillae formation, we carried out xenograft experiments using wild H460 cells (large cell lung carcinoma cell line) that constitutively expressed abundant NPC2 protein and NPC2 protein-depleted H460 cells by *NPC2 shRNA.* The xenografts of wild H460 cells and empty *shRNA* vector cells showed distinct papillae formation, whereas NPC2 protein-depleted H460 cells displayed markedly reduced or no papillae. Since all papillary tissues have open spaces we examined whether NPC2 protein might also contribute to the creation of open spaces. The TUNEL assay in the xenografts of wild and empty *shRNA* vector H460 cells showed massive cell death, and NPC2 protein-depleted cells displayed minimal cell death. Measurement of caspase 3/7 activities in cultured H460 cells supported NPC2 protein-mediated apoptotic cell death. The presence of excess NPC2 protein, however, did not always produce papillae as seen in the xenografts of CHO cells that were stably transfected with *NPC2.*

**Conclusions/Significance:**

The NPC2 protein of certain cells forms papillae coupled with apoptosis that creates open space. This protein may have future applications to modulate papillae formation and papillary growth in tumor tissues.

## Introduction

In humans, ovarian fimbria, choroid plexus of the brain, villi of the intestine, papillary cancer and papilloma are examples of papillary structure. Papillary structure consists of a fibrovascular core covered by epithelium creating nipple-like projections, called fronds. One unique feature of all papillary tissues is the presence of open spaces. There has been no report describing the mechanism of how papillae and open spaces are formed. We previously reported unique upregulation of *human epididymal protein-1* (*HE-1*) in papillary thyroid carcinoma by two-dimensional cDNA electrophoresis, an unbiased gene screening [1]. This gene was later named as *Niemann-Pick disease type C2* (*NPC2*) because mutation of this gene causes Niemann-Pick disease type 2C, a fatal cholesterol storage disorder of the lysosome [2], [3]. Our previous study suggested that the NPC2 could be a specific protein for human papillary structures [1]. In this study, all papillary tissues uniformly expressed NPC2 protein in the epithelium. Then, we hypothesized that the NPC2 protein could be involved in papillae formation. To examine this hypothesis, we carried out xenograft experiments using NPC2 protein-rich and-depleted H460 cells. Our present study revealed the requirement of NPC2 protein for papillary structure formation in certain cells. We describe here new functions of the NPC2 protein—papillae formation and creation of open spaces by apoptosis.

## Results

### Expression of NPC2 protein in papillary tissues

**Figure 1:**
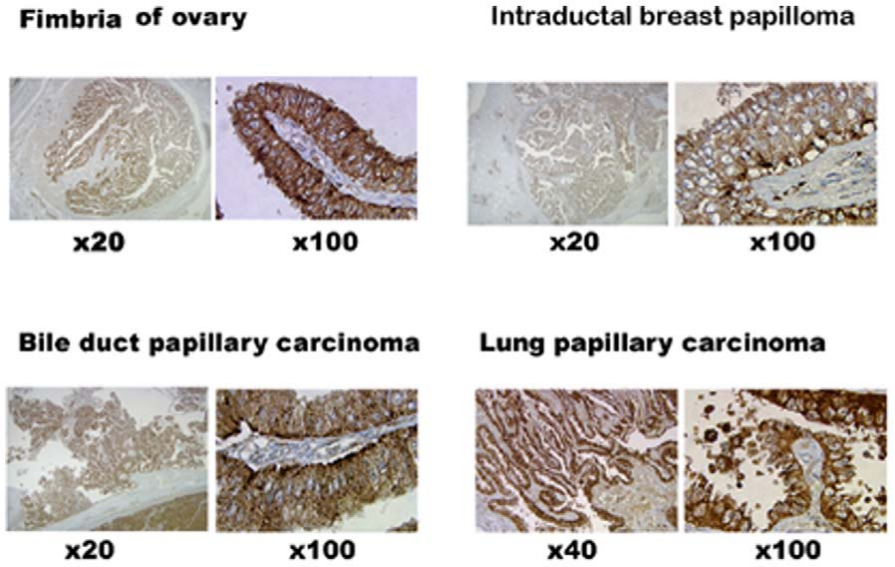
NPC2 protein expression in human papillary tissues. Immunohistochemical stain showed the presence of cytoplasmic NPC2 protein (brown color) in surgical samples of human papillary tissues. All papillary tissues have open spaces outside of papillary projections. The numbers indicate the degree of the magnification.

All papillary tissues examined so far showed cytoplasmic NPC2 protein in the epithelium as representative samples of normal papillae, papilloma and papillary cancer are shown in Figure 1. In addition, choroid plexus of the brain, colon polyps, and papillary carcinomas of breast expressed NPC2 protein (not shown here). Thus, NPC2 protein is associated with all papillary structures; this raised the possibility that NPC2 protein might contribute to papillae formation. To examine this hypothesis, we used NPC2 protein-rich and-depleted H460 cells.

### Characterization of wild H460 cells, empty vector and *NPC2-shRNA* containing H460 cells

**Figure 2:**
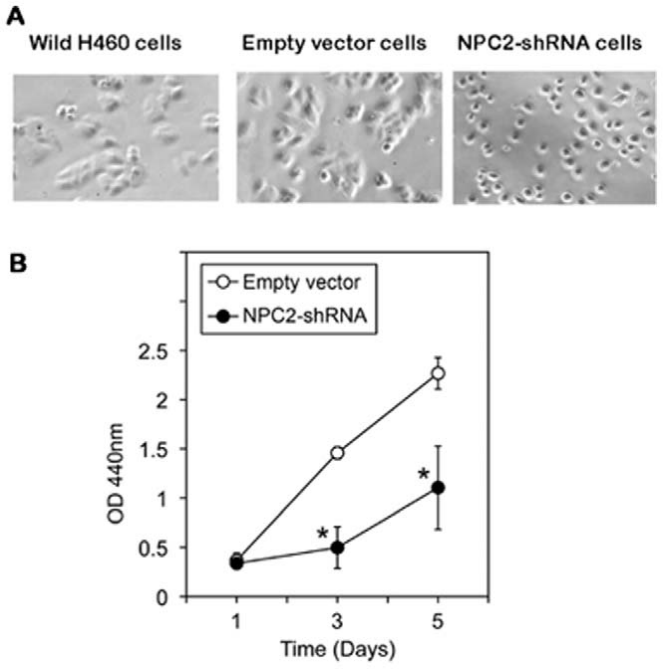
Characterization of three types of cultured H460 cells. Cell morphology: Wild and empty vector cells showed the same cell morphology, whereas cells containing *NPC2-shRNA* displayed clearly separated individual cells. Cell proliferation activity in NPC2-rich and-depleted H460 cells: Cells (1000 cells/well) were plated into a 96-well plate. Cell proliferation activity on each day was measured by incubating cells with the reagents for 2 hours in a CO2 incubator. The results are mean ± SD of quadruplicate samples of one of the three experiments. *p<0.01 when compared with the empty vector cells.

**Figure 3:**
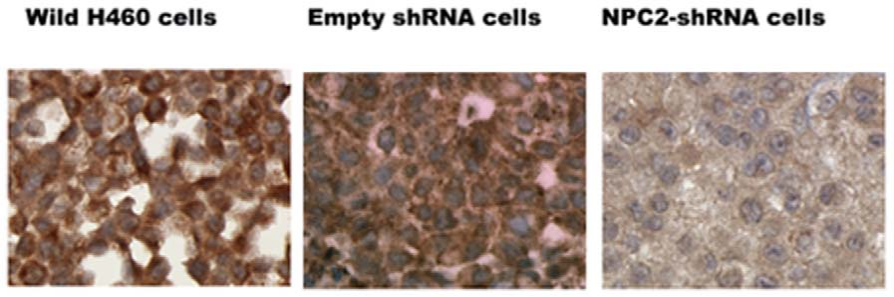
NPC2 protein expression in cultured H460 cells. Immunohistochemical staining showed abundant NPC2 protein in wild and empty vector cells and markedly reduced NPC2 protein in *NPC2-shRNA*-containing cells.

Wild H460 cells and empty *shRNA* vector H460 cells showed the same cell morphology; cells grew attaching each other. H460 cells containing *NPC2-shRNA* grew as separate and individual cells (Figure 2A). Cell proliferation activity in *NPC2-shRNA* containing cells was less than 50% of that of empty vector cells after the 3^rd^ day of culture (Figure 2B). Immunohistochemical stain showed abundant NPC2 protein in wild H460 cells and empty vector cells, whereas *NPC2-shRNA*-containing H460 cells had markedly reduced NPC2 protein expression (Figure 3).

### Papillae formation in xenografts

**Figure 4:**
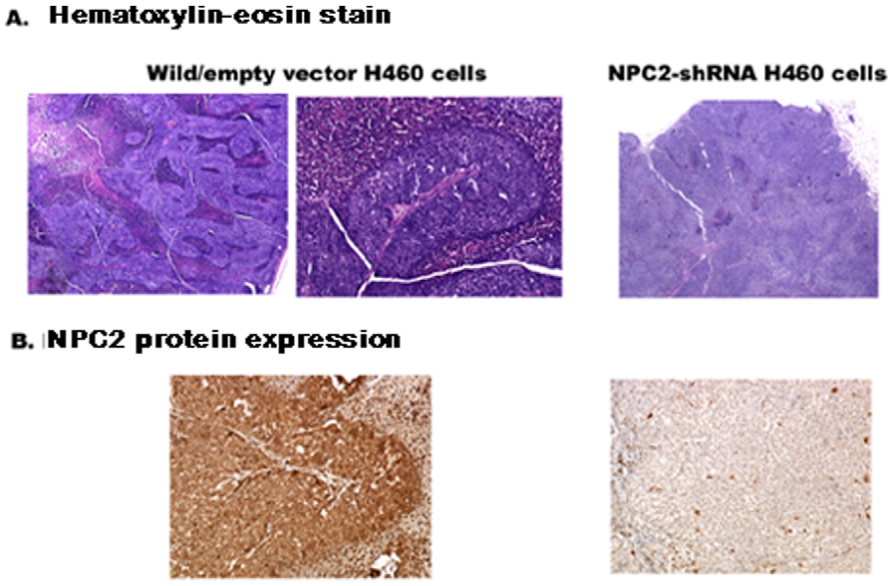
Papillae formation in xenografts of three types of H460 cells. A: Hematoxylin-eosin stain. B: Immunohistochemical stain for NPC2 protein. Distinct papillae formation was seen in wild and empty vector cells. No papilla was seen in *NPC2-shRNA* containing cells (A). NPC2 protein was localized in the epithelium of papillae (B).

We injected the three types of H460 cells (wild cells, empty vector cells, and *NPC2-shRNA*-containing cells) into nude mice and examined xenografts for papillae formation. The xenografts of wild H460 and empty vector cells showed distinct papillae formation, whereas NPC2-depleted H460 cells failed to show papilla (Figure 4A). The fibrovascular core covered by epithelium was clearly seen in the enlarged papilla. Immunohistochemistry showed localization of NPC2 protein in the entire epithelium of papillae, and little NPC2 protein was seen in NPC2-depleted cells as expected (Figure 4B).

### Requirement of apoptosis to form papillary shape

**Figure 5:**
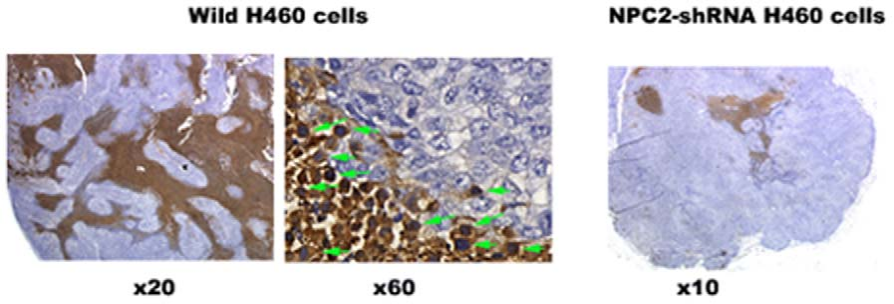
TUNEL assay in xenografts NPC2 protein-rich and-depleted cells. Apoptotic ell death was seen between papillary structures (brown colored areas) in wild H460 cells. The transition from epithelial cells (blue colored cells) to apoptotic cells (brown colored cells) was seen in the outer border of epithelium (middle panel). NPC2 protein-depleted H460 cells showed only small areas of apoptotic cell death.

**Figure 6:**
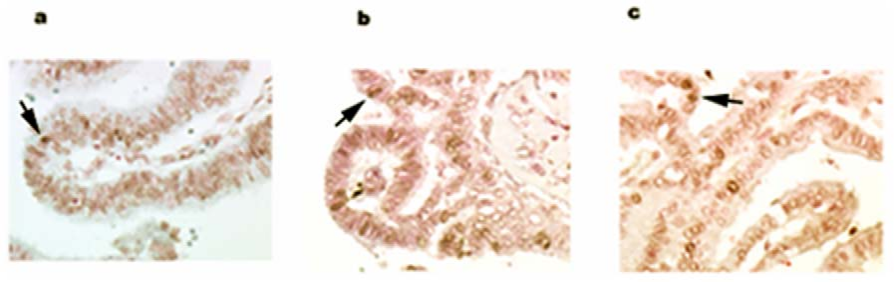
Presence of apoptotic cells in human papillary tissues. TUNEL assay showed the presence of apoptotic cells in representative samples of human papillary tissues; arrows indicate examples of apoptotic cells.

Papillary structures require open spaces between papillary fronds (Figure 1). We speculated that apoptosis might be responsible for creating these open spaces. The TUNEL assay from the xenograft of wild H460 cells showed massive cell death due to DNA fragmentation, whereas the xenograft of NPC2 protein-depleted H4607 cells had minimal cell death (Figure 5). Interestingly, the transition from epithelial cells to apoptotic cells was clearly seen in the outer border of epithelium (middle panel of Figure 5). To validate NPC2 protein-mediated apoptotic cell death, we measured caspase 3/7 activities from cultured H460 cells (empty vector cells and *NPC2-shRNA* containing cells); both cells were cultured under the identical conditions in the presence of hygromycin B. As shown in Table 1, caspase 3/7 activity in NPC2 protein-depleted cells was 13% of that of empty vector cells. Also, CHO cells stably transfected with *NPC2* showed a distinct caspase 3/7 activity expressed as relative luminescent unit (RLU) of 78×10^4^±15×10^4^ (mean ± SD of 3 determinations), whereas wild CHO cells had almost no caspase activity (RLU of 0 to 66). The presence of apoptotic cells was confirmed in surgical samples of human papillary tissues by the TUNEL assay (Figure 6).

**Table 1:** Caspase 3/7 activities in cultured H460 cells.

Cell types	Caspase 3/7 activities
Empty vector cells	100%±12.5%
NPC2-shRNA cells	13.5%±5.8%

Caspase activity of empty vector cell is considered as 100%. The results are mean ± SD of three different experiments performed in duplicates. *p<0.0004.

### Papillae formation by NPC2 protein-enriched CHO cells

**Figure 7:**
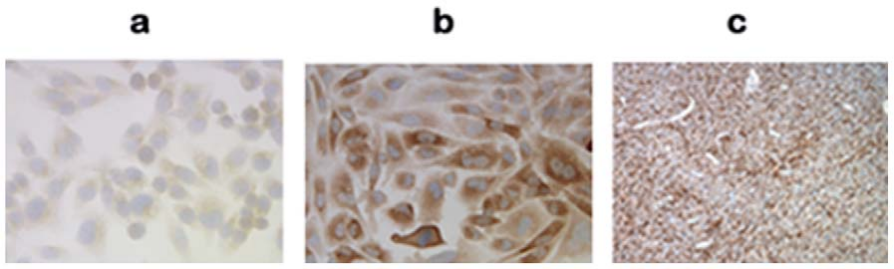
NPC2 protein expression in CHO cells. Immunohistochemical stain of NPC2 protein in wild CHO cell (a), CHO cells stably transfected with *NPC2* (b) and xenograft of CHO cells transfected with *NPC2* (c). No papilla was seen in the xenograft, despite abundant NPC2 protein expression.

We examined whether NPC2 protein-mediated papillae formation takes place regardless of cell types. Wild CHO cells showed very little NPC2 protein, and transfected cells displayed abundant NPC2 protein (Figure 7a, b). The xenograft of CHO cells stably transfected with *NPC2* failed to show visible papilla, despite abundant NPC2 protein expression (Figure 7c). Thus, excessive amount of NPC2 protein does not universally lead to papillae formation in some cells.

## Discussion

This study is focused on the mechanism of papillae formation, an unexplored area. Surgical specimens of all papillary tissues examined so far expressed NPC2 protein in the epithelium of papillae without exception (Figure 1). In fact, any form of papillary projections, such as intrafollicular papillary projection in goiter tissues [1] and colon polyps expressed NPC2 protein. Why is NPC2 protein present in all papillary tissues? We speculated that NC2 protein might be involved for formation of papillae. To demonstrate NPC2-mediated papillae formation in xenografts, cells expressing abundant NPC2 protein and depleted NPC2 protein are required. After screening several cancer cell lines, we found wild H460 cell line that constitutively expressed abundant NPC2 protein (Figure 3). To deplete *NPC2* in H460 cells, we constructed *shRNA* for *NPC2*, and our selected clone showed persistent and profound depletion of NPC2 protein (Figure 3). The xenografts of wild H460 cells and empty *shRNA* vector cells showed many papillae, whereas NPC2-depleted cells showed markedly reduced or no papillae formation depending on the microscopic fields examined (Figure 4). We attempted to create different levels of NPC2 protein expression by mixing wild H460 cells and *NPC2-shRNA* containing H460 cells with 1∶1 or 1∶5 ratios (wild cells: *NPC2-shRNA* treated cells). The xenografts of the mixed cells always showed many papillae as seen in the xenograft of wild H460 cells probably due to overwhelming growth of wild H460 cells over *NPC2-shRNA* containing cells. Decreased cell proliferation activity by *NPC2-shRNA* containing cells (Figure 2B) supports this phenomenon. Restricted localization of NPC2 protein to the epithelium suggests a specific role for NPC2 protein in papillary fronds formation. One unique feature of all papillary tissues is the presence of open spaces (Figure 1), and this could be crucial for the growth of papillary fronds. The mechanism of creating these open spaces has never been addressed. The TUNEL assay in the xenografts of wild H460 cells showed massive cell death between papillary fronds and a minimal cell death in NPC2 protein-depleted cells (Figure 5). Apoptosis is defined by caspase-mediated cell death [4]. Measurement of caspase 3/7 activities in cultured H460 cells supports NPC2 protein-mediated apoptosis (Table 1). Also, the TUNEL assay from human samples showed apoptotic cells (Figure 6). Apoptosis in papillary tissues can be summarized as follows. 1) Epithelial cells producing NPC2 protein can become apoptotic. 2) Apoptosis starts from the outer border of the epithelium. 3) Apoptosis should contribute to the formation of open spaces in papillary structures. In addition, apoptotic activity was seen in CHO cells transfected with *NPC2* but not in wild CHO cells. The NPC2 protein is localized in the lysosome for cholesterol binding and transport [2], [3], and the lysosome can activate intrinsic and extrinsic pathways of apoptosis under certain conditions [5]. Our study suggests that NPC2 protein itself can cause apoptosis. The mechanism of triggering apoptosis by excessive NPC2 protein has yet to be studied.

The question can be raised whether the expression of abundant NPC2 protein alone is sufficient to form papillae regardless of cell types. We examined this question in CHO cells that were stably transfected with *NPC2*. No papilla formation was seen in the xenografts, despite abundant NPC2 protein (Figure 7C). This finding suggests that an increase in cholesterol binding to NPC2 protein and its transport to the membrane, well known function of NPC2, is not solely responsible for papillae formation. Similarly, human liver highly expresses *NPC2* without papillary structure [2]. Therefore, an enrichment of NPC2 protein alone does not always produce papillae in certain cells. It is conceivable that additional factors or proteins are required to form papillae together with the NPC2 protein. We had an opportunity to examine *NPC2* hypomorphous mouse expressing 0–4% of NPC2 protein; this mouse was kindly provided by Dr. Peter Lobel [6]. We found normal papillae in the choroid plexus of the brain and villi of the intestine in this mouse. Therefore, the discrepancy of papillae formation between xenograft experiments (tumor of *NPC2* depleted cells under the skin) and live animal model (born with *NPC2* defect) exists. It is speculated that this mouse model may have NPC2 independent mechanisms or might have developed alternative mechanisms to form papillae. In humans, patients with Niemann-Pick Disease type 2C represent human model of NPC2 protein depletion. However, there is no autopsy report examining papillary tissues in this disorder.

The clinical application of NPC2 protein in papillae forming diseases has yet to be identified. Upregulation of *NPC2* in human papillary thyroid cancer [1] and significant decrease in cell proliferation in *NPC2*-depleted H460 cells (Figure 2B) suggest that NPC2 protein may also control cell proliferation in papillary tumors. Whether blocking NPC2 action has any therapeutic value for papillary cancer has yet to be studied in vivo. Exogenous NPC2 protein is taken into cells via two mannose 6 phosphate receptors that are ubiquitously present in the membrane [7] and corrected the abnormality of cholesterol handling in fibroblasts from a patient with *NPC2* mutation [2]. Therefore, it is interesting to examine whether addition of exogenous NPC2 protein restores disrupted papillary structures, such as epithelial atrophy of choroid plexus in Alzheimer disease [8].

## Methods

### Ethical statement

Paraffin sections of surgical samples were obtained from Hirosaki University Hospital, Hirosaki, Japan. The ethical committee of Hirosaki University School of Medicine exempted this human study including obtaining the consent from patients because paraffin section samples are 13 year-old with unidentifiable patients and no direct linkage of this study to the health care management. This is in accord with the human study guidelines established by the Ministry of Education and Science and the Ministry of Health and Welfare in Japan.

The animal experiments proposed here were fully approved by the GLA-VA Institutional Animal Care and Use Committee (IACUC) with protocol number of 02004-05. All animal experiments were carried out under the regulation of IACUC.

### Cells and surgical samples

H460 cells (large cell lung cancer cell line) and CHO cells (Chinese ovarian cells) were obtained from ATCC, Manassas, VA., and cultured in RPMI1640 medium and F-12 medium, respectively, in the presence of 10% fetal bovine serum and antibiotic cocktail.

### Preparation for NPC2 protein-rich and-depleted cells

We used wild H460 cells, since this cell line constitutively expressed abundant NPC2 protein. We also made NPC2 protein-rich CHO cells by stable transfection of CHO cells with *NPC2*. For the *NPC2* transfection experiment, we obtained the entire coding region of human *NPC2 cDNA* (NM_006432) from 3 µg total RNA of BHP 2-7 human papillary thyroid cancer cells by two-step RT-PCR using Superscript III (Invitrogen, CA.), Expand High Fidelity PCR System (Roche Applied Science, Indianapolis, IN), and custom primers (Invitrogen), 5′-GATGCGTTTCCTGGCAGCTA-3′ and 5′-CTGCTAACCAAGTGCTGCAT-3′. We introduced the amplicons into the *pCR 2.1* vector (Invitrogen), confirmed the correct sequences of the insert, and sub-cloned it into a mammalian expression vector *pcDNA3* (Invitrogen) with *Hind*III and *Xba*I (New England Biolabs, Ipswich, MA). We carried out transfection of CHO cells with *NPC2* in *pcDNA3* using Effectene Transfection Reagents (Qiagen, Valencia, CA) and selected clones expressing constant and abundant NPC2 protein in the presence of geneticin. Wild CHO cells contained very little NPC2 protein.

### *NPC2* knockdown experiments in H460 cells

We designed the target DNA sequences corresponding to *NPC2-shRNA* using the online program (Applied Biosystems/Ambion, Austin, TX) and ligated the target DNA to the *pSilencer3.1-H1 hygro* vector (Applied Biosystems/Ambion). After confirming the correct sequences of the insert from the amplified plasmid, we transfected H460 cells with *NPC2-shRNA* or *shRNA* vector alone (empty vector) using the T-20 program of the Amaxa Nucleofector II (Lonza Group, Ltd., Boston, MA). We selected stably transfected clones by culturing cells in the presence of 500 µg/ml hygromycin B (Invitrogen). The silencing effect of *NPC2-shRNA* on *NPC2* and its protein expression was confirmed by RT-PCR and immunohistochemistry staining, respectively. The most effective *NPC2*-*shRNA* insert inhibiting NPC2 protein expression contained the following sequences: 5′GATCCGTGGTATCCAGTGATTCGTTCTCAAGAGAAACGAATCACTGGATACCATTTTTTGGAAA-3′, and 5′AGCTTTTCCAAAAAATGGTATCCAGTGATTCGTTTCTCTTGAGAACGAATCACTGGATACCACG-3′.

### Animal experiments

We obtained female nude mice (15 g) from Charles-River Laboratories INC, Wilmington, MA, and all animals were kept in sterile cages. We injected 10^6^ cells in 0.1 ml phosphate buffered saline (PBS) subcutaneously to the flank region of mice. After tumors grew to 1–1.5 cm in diameter, we sacrificed animals in a CO2 chamber, removed tumors, and kept sliced tumor samples in formalin for immunostaining.

### Immunohistochemical staining, TUNEL assay and image analysis

Immunohistochemical studies were performed on formaldehyde-fixed, paraffin-embedded tissues. Deparaffinized sliced sections were incubated for 60 min at room temperature with the rabbit polyclonal anti-human NPC2 antiserum (1∶1000). Polyclonal anti-human NPC2 antibody was kindly provided by Dr. Peter Lobel, Rutgers University. Immunostaining was carried out using a Dako autostainer in conjunction with the EnVision™ detection kits (Dako, Carpinteria, CA). Positive controls (human epididymis tissue originally shown to express NPC2) and negative controls (without antibody) were used for NPC2 immunostaining. For TUNEL assay in sliced tissues, we used Apop Tag Peroxidases In Site Apoptosis Detection kit (Millipore Co., Bellireca, MA). For image analysis, we used Metamorph™ image analysis software version 7.5 (Molecular devices, Downingtown, PA).

### Caspase activity assay

Caspase 3/7 activities were measured from cultured cells using Promega caspase-Glo luminescent method (Promega, Madison. WI). We used H460 cells containing empty *shRNA* vector and *NPC2-shRNA*. These cells were thawed on the same day and cultured in 6-well plate in the presence of hygromycin B until 80% confluence was achieved. Cells were washed with PBS twice and sonicated in 1 ml PBS. An aliquot of 50 µl sonicate corresponding to 6×10^4^ cells was used immediately for caspase 3/7 assay. Each assay was run with or without 5 units Z-VAD-FMK, caspase inhibitor (Promega), by incubating the reaction mixture for 30 min at 37°C, and relative luminescent unit (RLU) was measured by GloMax 20/20 luminometer (Promega). We defined caspase activity as the RLU that was inhibited in the presence of caspase inhibitor. Similarly, we measured caspase3/7 activities from wild CHO cells and transfected CHO cells with *NPC2*.

### Cell proliferation assay

We used empty vector cells and *NPC2-shRNA* cells by plating 1000 cells/well in a 96-well plate; they were cultured under the identical condition in the presence of hygromycin B. Cell proliferation was measured using a quick cell proliferation assay kit (BioVision, Mountain View, CA) on first, third and fifth days of cell culture. The optical density from each well was measured at 440 nm using a microplate reader (Molecular devices, Sunnyvale, CA).

### Statistical analysis

The results of caspase activity and cell proliferation assay were analyzed by Student's *t*-test based on difference of variance (F test). The *p* value less than 0.05 were considered significant.
